# The Design of a New Catheter for Transcervical Artificial Insemination in Ewes

**DOI:** 10.3390/ani11123348

**Published:** 2021-11-23

**Authors:** Laura Falchi, Maria Teresa Zedda, Salvatore Pau, Mauro Ledda, Valentino Melosu, Salvatore Pier Giacomo Rassu

**Affiliations:** 1 Sezione di Clinica Ostetrica e Ginecologia, Dipartimento di Medicina Veterinaria, Università degli Studi di Sassari, Via Vienna n. 2, 07100 Sassari, Italy; zedda@uniss.it (M.T.Z.); nuvola@uniss.it (S.P.); vetleddamauro@gmail.com (M.L.); valentinomelosu@gmail.com (V.M.); 2 Sezione di Scienze Zootecniche, Dipartimento di Agraria, Università degli Studi di Sassari, Viale Italia n. 39, 07100 Sassari, Italy; pgrassu@uniss.it

**Keywords:** fertility, cervix, ovine, insemination gun, TCAI, frozen–thawed semen

## Abstract

**Simple Summary:**

In ovine species, transcervical artificial insemination is not easy to apply, due to the tortuous lumen of the cervix that does not allow the passage of routinely used catheters. Moreover, the quality of frozen–thawed semen in small ruminants is poor and these factors negatively affect the wide spreading of superior genotypes. The aim of this study was therefore to preliminarily test three newly designed insemination catheters, with bent tips of different lengths, in terms of reproductive performances in pluriparous ewes inseminated with frozen–thawed semen. Afterwards, the outcomes of insemination with the best performing catheter were compared to those obtained in ewes previously submitted to surgical incision of cervical folds, a technique that allows transcervical intrauterine deposition of semen. The results obtained indicated that a catheter with a bent tip of 5.0 mm allowed deep and fast intrauterine insemination, leading to pregnancy rates similar to those obtained following surgical incision of the folds. Further tests on the efficiency of the catheter are needed in field conditions and on a larger number of animals to assess the feasibility of the method in wide commercial insemination campaigns.

**Abstract:**

In ovine species, transcervical artificial insemination (TCAI) is limited by the poor quality of frozen–thawed semen and by the convoluted cervical lumen hampering the passage of inseminating devices. The aim of the study was to test the efficiency of three newly designed catheters with bent tips of 3.5 mm, 5.0 mm or 8.0 mm in terms of reproductive performances (experiment 1) and to compare the results of TCAI with the best performing catheter of experiment 1 to those obtained in ewes submitted to surgical incision of cervical folds (SICF) prior to insemination (experiment 2). The following parameters were assessed: time to pass the cervix; depth of cervical penetration; site of deposition of semen; pregnancy (PR); and lambing rates (LR). The results of experiment 1 indicated that the 5.0 mm tip catheter resulted in deeper and faster TCAI and higher PR and LR compared to 3.5 mm and 8.0 mm tip catheters (*p* < 0.05). In experiment 2, TCAI with the 5.0 mm catheter did not differ from TCAI after SICF in terms of depth of semen deposition, time to pass the cervix, PR and LR (*p* < 0.05). In conclusion, the use of a catheter that allowed transcervical uterine deposition of semen without excessive manipulation led to satisfactory pregnancy rates.

## 1. Introduction

The ovine cervix is characterized by a very narrow and misaligned lumen delimited by funnel-shaped folds that protrude caudally and are often in an eccentric position [1,2]. This peculiar anatomy makes the passage of an artificial insemination catheter difficult, often almost impossible, and contributes to the limited diffusion of transcervical artificial insemination (TCAI) in the ovine breeding system. Moreover, it needs to be pointed out that, apart from the cervical barrier, another significant restricting factor is represented by the low resistance of ram semen to cryopreservation. In ovine species, a relationship between the depth of penetration of the cervical lumen and the fertility rates achieved was reported in 1994 by Eppleston et al. [3]. The site of deposition of frozen–thawed sperm deep in the cervix (beyond the 3rd–4th fold) leads to consistently higher fertility rates compared to deposition at 1 cm depth in the lumen [3]. These observations are supported by the poor pregnancy rates obtained when frozen–thawed semen is deposited in the external os of the cervix, ranging from less than 5% [4,5] to 36% [6]. The problems related to the cervical barrier and the poor fertilizing ability of frozen–thawed semen in ovine artificial insemination have so far been overcome by laparoscopic AI (LAI). This technique has been widely used in recent decades since it allows direct uterine deposition of frozen–thawed semen via laparoscopy. The fertility rates range from 60% to 70% [5,7,8,9]. Nevertheless, it needs trained veterinarians, anesthesia and expensive equipment to be performed and it should be considered as provisional until an efficient TCAI technique is developed (as reviewed by [10]).

Basically, an optimal TCAI should: (i) allow deep cervical or uterine deposition of semen; (ii) be easy and fast to perform; (iii) be respectful of animal welfare; (iv) require inexpensive instruments; and (v) be applicable in field conditions.

In the past, different approaches have been attempted to improve the performances of TCAI but, although some of them appear convincing and successful in terms of fertility rates, none has had a wide in-field application. Recently, our research group presented a technique for surgical incision of cervical folds (SICF) that allowed us to achieve fertility rates comparable to those obtained with LAI [9,11] using frozen–thawed semen. Although it is a promising technique, to be performed once in the lifetime of the animal, it is a surgery that requires veterinarian skills and, as suggested by the authors, it should be devoted to small nuclei of selected high-value ewes. Among other methods, the use of drugs (hormones [12,13,14,15,16,17,18], chemokines [19], myorelaxants [20], hyaluronan [21], beta-adrenergic blocking agents [22]) and the design of inseminating catheters [23,24,25] associated with manipulation of the cervical canal (i.e., Guelph System [26,27]) have been investigated. Since hormones are in general expensive and, according to consumer demand, animal production should slowly move towards drug-free alternatives [28], the focus on the design of devices that could trespass the cervical lumen with no traumas on surrounding tissues could be the key to overcome the limitations of TCAI. Therefore, the primary objectives of the present study were to evaluate the reproductive performances of ewes inseminated with frozen–thawed semen in two sets of experiments: (1) preliminary testing of three different devices for transcervical insemination and (2) comparing two different TCAI techniques (i.e., the newly designed device and transcervical insemination following SICF).

## 2. Materials and Methods

### 2.1. Experimental Design

**Figure 1:**
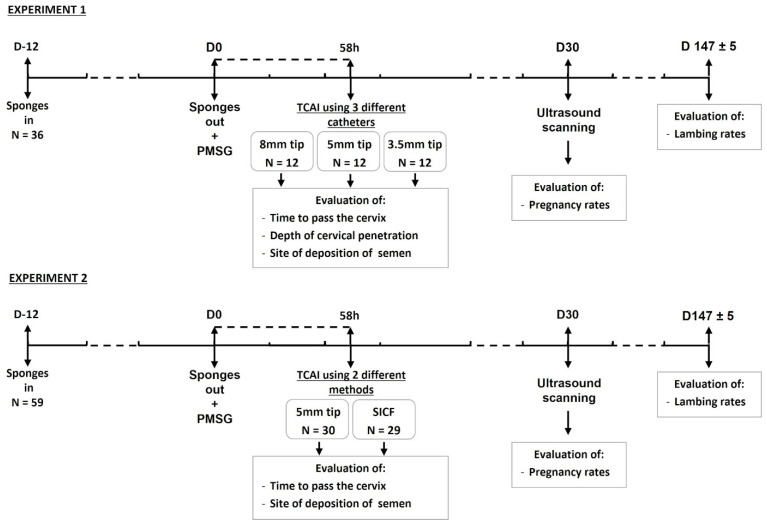
Design of the two experiments. Abbreviations: N = number; D = day; TCAI = transcervical artificial insemination; SICF = surgical incision of cervical folds.

All experimental procedures were carried out under European regulations on the care and welfare of animals in research and were ethically approved by the Organization in Charge of Animal Welfare and Animal Testing (Organismo Preposto al Benessere Animale ed alla Sperimentazione sugli Animali—OPBSA) of the University of Sassari (protocol number: 26064). All animals were fed hay and concentrates, and water was provided ad libitum. The experimental design is represented in Figure 1. A total number of 95 pluriparous Sarda ewes (3–4 years old and about 45 kg live weight) were randomly assigned to two different experiments. In experiment 1 (*n* = 36), three different catheters for TCAI were tested, and in experiment 2 (*n* = 59), two different TCAI methods, insemination with the best performing catheter of experiment 1 and insemination following surgical incision of the cervical folds (SICF), were compared.

### 2.2. Estrous Synchronization

In all experimental animals, synchronization of estrous cycles was achieved by inserting intravaginal progestagen sponges (Crono-gest 20 mg, Intervet Italia S.r.l, Segrate, Italy) pre-treated with antibiotic powder (Izoaspersorio, Izo, Brescia, Italy) for 12 days and injecting IM 300 IU of PMSG (Folligon, Intervet Italia S.r.l, Italy) at the time of sponge removal.

### 2.3. Semen Collection and Preparation

Ejaculates of three adult Sarda rams of proven fertility were collected by artificial vagina. Only samples having a concentration of at least 3 × 10^9^ spz/mL and mass motility ≥3 (score from 0 (no waves) to 5 (vigorous swirling waves)) were further processed. Semen was pooled and extended at 30 °C with Tris-EY (egg yolk, 20%) supplemented with 6% glycerol to reach a final concentration of 400 × 10^6^ spz/mL. Extended semen was gradually cooled from 30 °C to 4 °C in 5 h and loaded into 0.25 mL straws (insemination dose: 100 × 10^6^ spz/straw). Straws were then exposed for 7 min to LN_2_ (liquid nitrogen) vapors at 5 cm above the surface, plunged in LN_2_ and then stored until use. Post-thawing motility was assessed by CASA (Computer Assisted Sperm Analysis, IVOS, Hamilton Thorne, Biosciences) in a straw from each batch. Semen with ≥45% progressive motility was considered suitable for insemination. At the time of insemination, thawing was performed at 37 °C for 30 s.

### 2.4. Experiment 1

The experiment was carried out on 36 pluriparous Sarda ewes randomly allocated to three different groups (*n* = 12). Following synchronization of ovulation, 58 h after removal of the sponges, ewes were submitted to TCAI using three different insemination catheters. All the inseminations were performed by the same professional. 

#### Catheter Design

**Figure 2:**
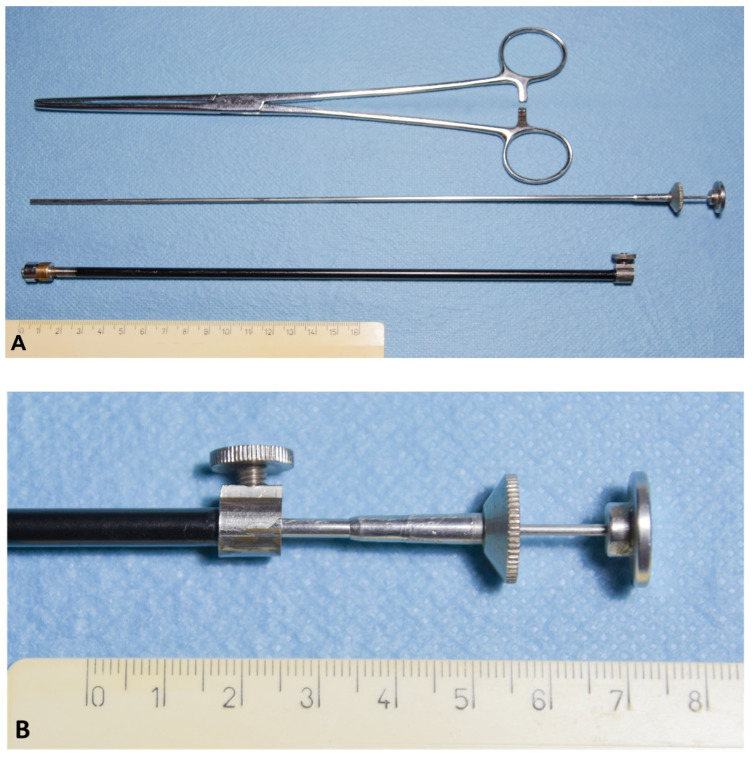

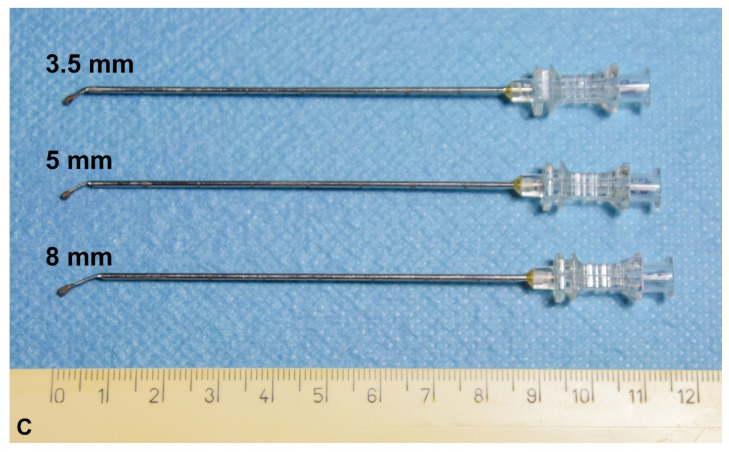
(**A**) Tools used for transcervical artificial insemination. From top to bottom: Bozeman forceps for cervical retraction; Cassou insemination gun for small ruminants (IMV technologies, France), inserted into the insemination catheter, composed of a stainless-steel tube (length: 28.5 cm, inner diameter: 3.5 mm); (**B**) detail of one end of the catheter fitted with a screw system to keep the Cassou gun secured; (**C**) detail of the other end composed of a modified epidural needle (Ø 14 G) with a 45° bent rounded atraumatic tip of different lengths: 3.5 mm, 5.0 mm or 8.0 mm.

Catheters were composed of a stainless-steel tube 28.5 cm long with an inner diameter of 3.5 mm, containing a Cassou insemination gun for small ruminants (IMV Technologies, L’Aigle, France) fitting a sanitary sheath (Figure 2A). One end of the catheter had a screw system that kept the Cassou gun secured (Figure 2B), while the other end was composed of a modified epidural needle (Ø 14 G) with a 45° bent rounded atraumatic tip of different lengths: 3.5 mm, 5.0 mm or 8.0 mm (Figure 2C).

### 2.5. Experiment 2

The experiment was carried out on 59 ewes allocated to two different groups: *n* = 30 were inseminated using the catheter that in experiment 1 yielded the best performances; *n* = 29 were previously submitted to two incisions of cervical folds [9,11] and transcervically inseminated. These ewes were the same subjects used for the study published by Pau et al. (2019) [11]. Briefly, surgery was performed within 24 h from lambing and after confirming expulsion of fetal membranes. Following mild sedation and epidural anesthesia, ewes were placed in dorsal recumbency with the hindquarters slightly elevated, and the external os of the cervix was localized by the aid of a speculum inserted in the vagina. The most external fold of the os was grasped with a Duval forceps and the whole cervix, fold by fold, was gently retracted caudally up to the vaginal vestibulum until complete exteriorization. Afterwards, each fold was incised dorsally and ventrally. Local antibiotic treatment was provided, and the extruded folds were gently repositioned. Ewes were kept under post-operatory observation for 24 h.

### 2.6. Ewe Restraint and Cervical Manipulation for TCAI

For transcervical insemination, animals were placed in a cradle in dorsal recumbence and the vulva and perineal area were carefully rinsed. The cervix was located by the insertion of a vaginal speculum fitted with a light source and the external fold of the os was grasped with the aid of Bozeman forceps. The cervix was then gently retracted caudally and positioned to allow proper manipulation with the modified tip fitted on the stainless- steel tube. During cervical manipulation, semen was thawed and loaded on the Cassou gun. Once the site of deposition of semen was reached, the gun was inserted into the stainless-steel tube and secured, and insemination was carried out. Ewes previously submitted to SICF were inseminated using a commercial AI catheter for small ruminants (Cassou mini-pistolet for ovine-caprine, IMV Technologies, France).

### 2.7. Data Collection

**Figure 3:**
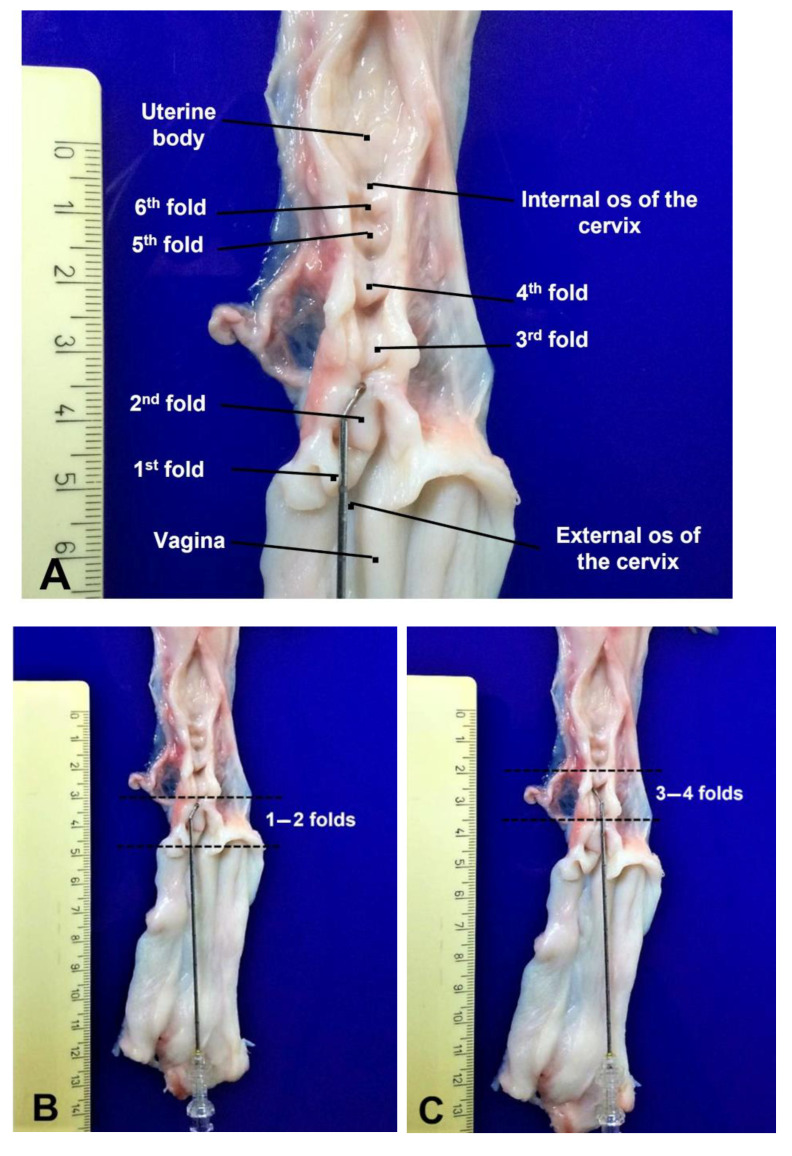

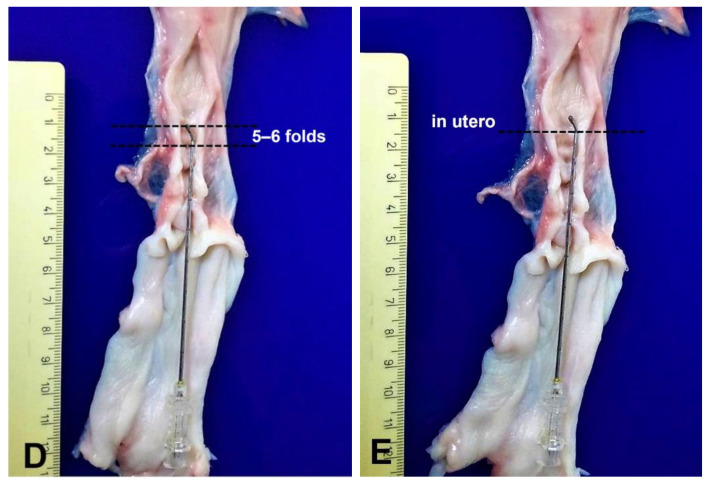
(**A**) Cervix of a pluriparous Sarda ewe characterized by 5–6 cervical folds that protrude into the cervical lumen; position of the 5 mm tip catheter when semen was deposited beyond (**B**) the first two folds; (**C**) the 3rd–4th fold; (**D**) the 5th–6th fold; (**E**) in utero.

In experiment 1, the depth of penetration through the cervical lumen was assessed by reporting the number of cervical folds overcome by the three catheters (1st–2nd folds; 3rd–4th folds; 5th–6th folds; in utero; Figure 3A–E). For both experiments, the time taken to trespass the cervical lumen was recorded and data about site of deposition of semen (in cervix vs. in utero) were collected. Pregnancy rates (pregnant ewes/inseminated ewes) were assessed by transrectal ultrasound (MyLab One, Esaote, Genova, Italy) on day 30 after insemination and lambing rates were also recorded.

### 2.8. Statistical Analyses

Collected data were statistically analyzed using STATA 11.2/IC (StataCorp LP, College Station, TX, USA) by the chi-square test or Fisher’s exact test to determine the effect of the three catheters (experiment 1) and of the two insemination techniques (experiment 2) on depth of cervical penetration, site of deposition of semen and pregnancy and lambing rates. The significance level was defined for *p* < 0.05.

## 3. Results

### 3.1. Experiment 1

The results of experiment 1 are summarized in Table 1, Table 2 and Table 3. The device used for TCAI had a significant effect on all the measured parameters (i.e., time to reach the uterus, depth of semen deposition, pregnancy and lambing rates; *p* < 0.05). In general, TCAI with the 5.0 mm tip catheter resulted in higher pregnancy and lambing rates compared to the 8.0 mm tip catheter (*p* < 0.05), while there was no significant difference compared to the 3.5 mm tip catheter (Table 1; *p* > 0.05). However, the 5.0 mm tip catheter allowed deeper and faster deposition of semen compared to the other two devices (Table 2 and Table 3; *p* < 0.05).

The site of deposition of semen, measured by the number of overcome cervical folds, had a significant effect on pregnancy outcomes (*p* < 0.05). Most of the ewes inseminated with the 5.0 mm tip catheter directly in the uterine lumen were pregnant (77.7%), while none of those that had semen deposition in cervical lumen were pregnant. Using the 8.0 mm tip catheter, the only pregnant ewes had semen deposited beyond the 3rd–4th cervical fold. Deposition of semen with the 3.5 mm tip catheter led to 16.6% pregnant ewes, depositing semen beyond the 5th–6th cervical fold (*n* = 1) and in utero (*n* = 1) (Table 2).

The time taken to reach the cervical lumen affected pregnancy and lambing rates (*p* < 0.05). Overall, when insemination took more than 60 s, pregnancy rates were significantly lower (*p* < 0.05). In most of the ewes inseminated with the 8.0 mm (91.6%) or the 3.5 mm (75%) tip catheter, the procedure lasted more than 60 s and led to low pregnancy rates (9% and 11%, respectively). Conversely, insemination with the 5.0 mm tip catheter allowed faster semen deposition: 33.3% of ewes were inseminated in less than 10 s and most of them (3/4 ewes) were pregnant and lambed regularly (Table 3).

### 3.2. Experiment 2

Results of experiment 2 are summarized in Table 4, Table 5 and Table 6. In general, no significant differences in pregnancy and lambing rates were found between ewes inseminated with the 5.0 mm tip catheter and those inseminated after SICF (Table 4; *p* > 0.05).

Time taken to reach the cervical lumen was not affected by the method of insemination (Table 5; *p* > 0.05). Reaching the cervical lumen with both insemination methods took less than 30 s in most of the animals. In four ewes submitted to SICF, semen was deposited in the cervix as it was impossible for the catheter to reach the uterine lumen in <60 s, resulting in one pregnancy (Table 6). Using the 5.0 mm tip catheter, in four ewes, semen was deposited cervically in >60 s, resulting in no pregnancies.

## 4. Discussion

The results presented in this report indicate that the design of an insemination catheter with a bent tip of 5.0 mm was successful in allowing deeper and faster transcervical insemination with frozen–thawed semen in Sarda ewes. The pregnancy and lambing rates obtained using this catheter were comparable to those obtained by insemination following SICF and, in most of the ewes, semen deposition occurred in the uterus. In ewes submitted to SICF, the overlapping of cervical folds that hampers the passage of routinely used inseminating devices is avoided by dorsal and ventral incisions of each fold. This procedure allows a smooth and atraumatic penetration of the cervical lumen and intrauterine insemination, obtaining pregnancy rates that are easily comparable to those deriving from laparoscopic AI [9]. We have sufficient evidence in the literature demonstrating that in ovine species, the closer the semen is deposited to the site of fertilization, the higher the pregnancy rates [3]. This is supported by the satisfactory pregnancy and lambing rates obtained by laparoscopic AI [3] or by TCAI following SICF [9,11]. With both techniques, the cervical barrier is bypassed, and the deposition of frozen–thawed semen is intrauterine. However, these techniques are invasive and require anesthesia, proper training of the operator is mandatory and, in our opinion, they should be gradually destined to limited nuclei of animals for genetic selection. Nevertheless, the influence of the depth of semen deposition by TCAI on pregnancy rates has been questioned by several authors. Among others, Kumar et al. [29] reported no correlation among depth of insemination with frozen–thawed semen on pregnancy and lambing rates.

In the literature, several research groups designed catheters for enhancing fertility rates following TCAI and, among them, many authors reported that using catheters with bent tips allowed deeper penetration compared to straight ones [2,23,26]. Alvarez et al. [23] used a catheter with an eccentric bent tip of 6.0 mm, similar to the one used in the present experiment, and reported pregnancy rates of around 45% using chilled semen. In contrast, our results showed that inseminating ewes using the catheter with the 5.0 mm bent tip led to higher pregnancy rates (around 63%), although frozen–thawed semen was used. Besides small differences in the design of the catheters, the discrepancies in the results could be attributable primarily to the different techniques used to perform the insemination: the gentle cervical retraction exerted in the present experiments allowed deep penetration and semen deposition. Alvarez et al. [23] did not use this technique and reported semen deposition as deep as possible in the cervical lumen. Moreover, breed differences in the weight and size of the animals chosen for the experiments may have influenced the morphometry of the cervical lumen, significantly affecting the depth of cervical penetration [2,12,30,31]. Another crucial factor affecting the outcomes of TCAI in ewes is the age of the inseminated animals, more precisely intended as number of parturitions. Changes in the morphology and the complexity of the cervical lumen in mature pluriparous ewes have been previously described by several authors [1,2,31]. Prior to the present trials, the ewes lambed 3 to 4 times and we can therefore hypothesize that cervical folds underwent mechanical stretching, making the lumen less convoluted and allowing deeper passage of the inseminating catheter. 

A further advantage in the use of the newly designed catheter could be that cervical manipulation was carried out with the tip of the catheter fitted in the outer stainless-steel tube, and that the Cassou gun was inserted at a later stage, when the site of semen deposition (cervical lumen or uterus) was reached. In this way, semen was thawed and loaded in the Cassou gun during cervical manipulation. This procedure may have limited the decay of the quality of thawed semen, easily damaged by cryopreservation, shortening the time between thawing and deposition in the female genital tract.

The time taken to reach and possibly pass the cervical lumen is the indirect measure of the amount of manipulation of the cervix that, if prolonged, could potentially affect the success of the insemination method. Excessive cervical manipulation induced tissue damage, with release of local prostaglandins and recall of neutrophiles, altered the composition of cervical mucus and the uterine micro-environment, leading to pregnancy failure [23,32]. Therefore, the less the manipulation, the higher the chance of having pregnant animals. In both experiments, we observed that when cervical manipulation took more than 30 s, pregnancy rates were low, ranging from 8.7% to 29.0%. This was in agreement with what has been previously published. Alvarez et al. [23] reported successful pregnancy rates when TCAI was performed in less than 10 s and conversely, when penetration took more than two minutes, pregnancy rates dropped, and the technique becomes practically unfeasible [33,34,35]. 

## 5. Conclusions

The site of semen deposition and time taken to pass through the cervical lumen are essential parameters for the assessment of the feasibility of an insemination technique. In light of these observations, the design of a bent catheter with a 5.0 mm blunt tip allowed fast and deep transcervical insemination in pluriparous Sarda ewes, achieving satisfactory pregnancy rates. The results obtained are comparable to those obtained by techniques such as SICF or LAI that ensure uterine deposition of semen. However, further tests on the efficiency of the catheter are needed in field conditions and on a larger number of animals.

## Figures and Tables

**Table 1:** Pregnancy (PR) and lambing (LR) rates of ewes submitted to transcervical artificial insemination by three different catheters.

Insemination Catheter Type	Ewes	PR (%)	LR (%)
8.0 mm	12	1 (8.3) ^a^	1 (8.3) ^a^
5.0 mm	12	7 (58.3) ^b^	7 (58.3) ^b^
3.5 mm	12	2 (16.6) ^ab^	2 (16.6) ^ab^
Total	36	10 (27.7)	10 (27.7)

Different superscripts (^a,b^) indicate within column significant differences for *p* < 0.05.

**Table 2:** Pregnancy (PR) and lambing (LR) rates in relation to the site of semen deposition in the cervical lumen using three different catheters.

Insemination Catheter Type	Site of Semen Deposition											
	1–2 Folds			3–4 Folds			5–6 Folds			In Utero		
	Ewes	PR (%)	LR (%)	Ewes	PR (%)	LR (%)	Ewes	PR (%)	LR (%)	Ewes	PR (%)	LR (%)
8.0 mm	2	0 (0)	0 (0)	5 ^a^	1 (20.0)	1(20.0)	4	0 (0)	0 (0)	1 ^a^	0 (0) ^a^	0 (0) ^a^
5.0 mm	0	-	-	0 ^b^	-	-	3	0 (0)	0 (0)	9 ^b^	7 (77.8) ^b^	7 (77.8) ^b^
3.5 mm	0	-	-	4 ^a^	0 (0)	0 (0)	5	1 (20.0)	1(20.0)	3 ^a^	1 (33.3) ^a^	1 (33.3) ^a^
Overall	2	0 (0) ^x^	0 (0) ^x^	9	1 (11.1) ^x^	1 (11.1) ^x^	13	1 (7.7) ^x^	1 (7.7) ^x^	12	8 (66.7) ^y^	8 (66.7) ^y^

Different superscripts indicate within column (^a,b^) and within row (^x,y^) significant differences for *p* < 0.05.

**Table 3:** Pregnancy (PR) and lambing (LR) rates in relation to the time taken to reach the cervical lumen using three different catheters.

Insemination Catheter Type	Time to Reach the Cervical Lumen											
	<10 s			10–30 s			30–60 s			>60 s		
	Ewes	PR (%)	LR (%)	Ewes	PR (%)	LR (%)	Ewes	PR (%)	LR (%)	Ewes	PR (%)	LR (%)
8.0 mm	0 ^a^	0 (0)	0 (0)	1	0 (0)	0 (0)	0	0 (0)	0 (0)	11 ^a^	1 (9.1)	1 (9.1)
5.0 mm	4 ^b^	3 (75.0)	3 (75.0)	3	3 (100)	3 (100)	2	1 (50.0)	1 (50.0)	3 ^b^	0 (0)	0 (0)
3.5 mm	1 ^ab^	1 (100)	1 (100)	0	0 (0)	0 (0)	2	0 (0)	0 (0)	9 ^a^	1 (11.1)	1 (11.1)
Overall	5	4 (80.0) ^x^	4 (80) ^x^	4	3 (75.0) ^xy^	3 (75.0) ^xy^	4	1 (25.0) ^yz^	1 (25.0) ^yz^	23	2 (8.7) ^z^	2 (8.7) ^z^

Different superscripts indicate within column (^a,b^) and within row (^x,y,z^) significant differences for *p* < 0.05.

**Table 4:** Pregnancy (PR) and lambing (LR) rates of ewes inseminated with two different methods for transcervical artificial insemination (TCAI).

Transcervical Artificial Insemination Method	Ewes	PR (%)	LR (%)
Catheter 5.0 mm tip	30	19 (63.3)	16 (53.3)
Surgical incision of cervical folds	29	23 (79.3)	21 (72.4)
Overall	59	42 (71.2)	37 (62.7)

**Table 5:** Pregnancy (PR) and lambing (LR) rates in relation to the time taken to reach the cervical lumen using two different methods for transcervical artificial insemination.

Transcervical Artificial Insemination Method	Time to Reach The Cervical Lumen											
	<10 s			10–30 s			30–60 s			>60 s		
	Ewes	PR (%)	LR (%)	Ewes	PR (%)	LR (%)	Ewes	PR (%)	LR (%)	Ewes	PR (%)	LR (%)
Catheter 5.0 mm tip	11	8 (72.7)	6 (54.5)	11	11 (100)	10 (90.9)	4	0 (0)	0 (0)	4	0 (0)	0 (0)
Surgical incision of cervical folds	17	14 (82.4)	14 (82.4)	6	6 (100)	5 (83.3)	5	3 (60.0)	1 (20.0)	1	1 (100)	1 (100)
Overall	28	22 (78.6)	20 (71.4)	17	17 (100)	15 (88.2)	9	3 (33.3)	1 (11.1)	5	1 (20.0)	1 (20.0)

**Table 6:** Pregnancy (PR) and lambing (LR) rates in relation to the site of semen deposition using two different methods for transcervical artificial insemination.

Transcervical Artificial Insemination Method	Site of Semen Deposition					
	In Cervix			In Utero		
	Ewes	PR (%)	LR (%)	Ewes	PR (%)	LR (%)
Catheter 5.0 mm tip	4	0 (0)	0 (0)	26	19 (73.1)	16 (61.5)
Surgical incision of cervical folds	4	1 (25.0)	1 (25.0)	25	22 (84.6)	20 (80.0)
Total	8	1 (12.5)	1 (12.5)	51	41 (80.4)	36 (70.6)

## Data Availability

Data will be made available upon reasonable request.

## References

[1] Kershaw C.M., Khalid M., McGowan M.R., Ingram K., Leethongdee S., Wax G., Scaramuzzi R.J. (2005). The anatomy of the sheep cervix and its influence on the transcervical passage of an inseminating pipette into the uterine lumen. Theriogenology.

[2] Kaabi M., Alvarez M., Anel E., Chamorro C.A., Boixo J.C., de Paz P., Anel L. (2006). Influence of breed and age on morphometry and depth of inseminating catheter penetration in the ewe cervix: A postmortem study. Theriogenology.

[3] Eppleston J., Salamon S., Moore N.W., Evans G. (1994). The depth of cervical insemination and site of intrauterine insemination and their relationship to the fertility of frozen-thawed ram semen. Anim. Reprod. Sci..

[4] Hiwasa M., Kohno H., Togari T., Okabe K., Fukui Y. (2009). Fertility after different artificial insemination methods using a synthetic semen extender in sheep. J. Reprod. Dev..

[5] Masoudi R., Zare Shahneh A., Towhidi A., Kohram H., Akbarisharif A., Sharafi M. (2017). Fertility response of artificial insemination methods in sheep with fresh and frozen-thawed semen. Cryobiology.

[6] Richardson L., Hanrahan J.P., Donovan A., Martí J.I., Fair S., Evans A.C.O., Lonergan P. (2012). Effect of site of deposition on the fertility of sheep inseminated with frozen-thawed semen. Anim. Reprod. Sci..

[7] Windsor D.P., Szell A.Z., Buschbeck C., Edward A.Y., Milton J.T., Buckrell B.C. (1994). Transcervical artificial insemination of Australian Merino ewes with frozen-thawed semen. Theriogenology.

[8] Fair S., Hanrahan J.P., O’Meara C.M., Duffy P., Rizos D., Wade M., Donovan A., Boland M.P., Lonergan P., Evans A.C. (2005). Differences between Belclare and Suffolk ewes in fertilization rate, embryo quality and accessory sperm number after cervical or laparoscopic artificial insemination. Theriogenology.

[9] Pau S., Falchi L., Ledda M., Pivato I., Valentino M., Bogliolo L., Ariu F., Zedda M.T. (2020). Reproductive Performance Following Transcervical Insemination with Frozen Thawed Semen in Ewes Submitted to Surgical Incision of Cervical Folds (SICF): Comparison with Laparoscopic Artificial Insemination. Animals.

[10] Alvarez M., Anel-Lopez L., Boixo J.C., Chamorro C., Neila-Montero M., Montes-Garrido R., de Paz P., Anel L. (2019). Current challenges in sheep artificial insemination: A particular insight. Reprod. Domest. Anim..

[11] Pau S., Falchi L., Ledda M., Bogliolo L., Ariu F., Zedda M.T. (2019). Surgery on cervical folds for transcervical intrauterine artificial insemination with frozen-thawed semen enhances pregnancy rates in the sheep. Theriogenology.

[12] Falchi L., Taema M., La Clanche S., Scaramuzzi R.J. (2012). The pattern of cervical penetration and the effect of topical treatment with prostaglandin and/or FSH and oxytocin on the depth of cervical penetration in the ewe during the peri-ovulatory period. Theriogenology.

[13] Khalifa R.M., Sayre B.L., Lewis G.S. (1992). Exogenous oxytocin dilates the cervix in ewes. J. Anim. Sci..

[14] Stellflug J.N., Wulster-Radcliffe M.C., Hensley E.L., Cowardin E.A., Seals R.C., Lewis G.S. (2001). Oxytocin-induced cervical dilation and cervical manipulation in sheep: Effects on laparoscopic artificial insemination. J. Anim. Sci..

[15] Wulster-Radcliffe M.C., Costine B.A., Lewis G.S. (1999). Estradiol-17 beta-oxytocin-induced cervical dilation in sheep: Application to transcervical embryo transfer. J. Anim. Sci..

[16] Akinbami M.A., Meredith S., Warren J.E., Anthony R.V., Day B.N. (1990). Cervical dilation, conception rate, and concentrations of progesterone and estradiol-17B in postpartum ewes treated with porcine relaxin. Theriogenology.

[17] Bartlewski P.M., Candappa I.B. (2015). Assessing the usefulness of prostaglandin E2 (Cervidil) for transcervical artificial insemination in ewes. Theriogenology.

[18] Leethongdee S., Khalid M., Bhatti A., Ponglowhapan S., Kershaw C.M., Scaramuzzi R.J. (2007). The effects of the prostaglandin E analogue Misoprostol and follicle-stimulating hormone on cervical penetrability in ewes during the peri-ovulatory period. Theriogenology.

[19] Croy B.A., Prudencio J., Minhas K., Ashkar A.A., Galligan C., Foster R.A., Buckrell B., Coomber B.L. (1999). A preliminary study on the usefulness of huIL-8 in cervical relaxation of the ewe for artificial insemination and for embryo transfer. Theriogenology.

[20] Santos K.C., Monte A.P., Lima J.T., Ribeiro L.A., Palheta Junior R.C. (2016). Role of NO-cGMP pathway in ovine cervical relaxation induced by Erythroxylum caatingae Plowman. Anim. Reprod. Sci..

[21] Perry K., Haresign W., Wathes D.C., Khalid M. (2010). Intracervical application of hyaluronan improves cervical relaxation in the ewe. Theriogenology.

[22] Gündüz M.C., Turna Ö., Cirit Ü., Uçmak M., Tek Ç., Sabuncu A., Bacınoğlu S. (2010). Lambing rates and litter size following carazolol administration prior to insemination in Kivircik ewes. Anim. Reprod. Sci..

[23] Álvarez M., Chamorro C.A., Kaabi M., Anel-López L., Boixo J.C., Anel E., Anel L., de Paz P. (2012). Design and “in vivo” evaluation of two adapted catheters for intrauterine transcervical insemination in sheep. Anim. Reprod. Sci..

[24] Macías A., Ferrer L.M., Ramos J.J., Lidón I., Rebollar R., Lacasta D., Tejedor M.T. (2017). Technical Note: A new device for cervical insemination of sheep—Design and field test. J. Anim. Sci..

[25] Wulster-Radcliffe M.C., Lewis G.S. (2002). Development of a new transcervical artificial insemination method for sheep: Effects of a new transcervical artificial insemination catheter and traversing the cervix on semen quality and fertility. Theriogenology.

[26] Halbert G.W., Dobson H., Walton J.S., Buckrell B.C. (1990). A technique for transcervical intrauterine insemination of ewes. Theriogenology.

[27] Halbert G.W., Dobson H., Walton J.S., Sharpe P., Buckrell B.C. (1990). Field evaluation of a technique for transcervical intrauterine insemination of ewes. Theriogenology.

[28] Scaramuzzi R.J., Martin G.B. (2008). The importance of interactions among nutrition, seasonality and socio-sexual factors in the development of hormone-free methods for controlling fertility. Reprod. Domest. Anim..

[29] Kadirvel G., Kumar S., Kumaresan A. (2009). Lipid peroxidation, mitochondrial membrane potential and DNA integrity of spermatozoa in relation to intracellular reactive oxygen species in liquid and frozen-thawed buffalo semen. Anim. Reprod. Sci..

[30] Abril-Parreño L., Krogenæs A.K., Byrne C.J., Donovan A., Stuen S., Caldas E., Diskin M., Druart X., Fair S. (2021). Ewe breed differences in cervical anatomy and cervicovaginal mucus properties: An international study. Theriogenology.

[31] El Khalil K., Allai L., Fatet A., Benmoula A., Hamidallah N., Badi A., Moussafir Z., Ibnelbachyr M., El Amiri B. (2018). Morphometry and depth of inseminating catheter penetration in prolific and non- prolific ewes at different ages: A post mortem study. Anim. Reprod. Sci..

[32] Rickard J.P., Pool K.R., Druart X., de Graaf S.P. (2019). The fate of spermatozoa in the female reproductive tract: A comparative review. Theriogenology.

[33] Windsor D.P. (1995). Factors influencing the success of transcervical insemination in Merino ewes. Theriogenology.

[34] Buckrell B.C., Buschbeck C., Gartley C.J., Kroetsch T., McCutcheon W., Martin J., Penner W.K., Walton J.S. (1994). Further development of a transcervical technique for artificial insemination in sheep using previously frozen semen. Theriogenology.

[35] Casali R., Pinczak A., Cuadro F., Guillen-Munoz J.M., Mezzalira A., Menchaca A. (2017). Semen deposition by cervical, transcervical and intrauterine route for fixed-time artificial insemination (FTAI) in the ewe. Theriogenology.

